# Relationship between oral frailty, health-related quality of life, and survival among long-term care residents

**DOI:** 10.1007/s41999-023-00859-x

**Published:** 2023-09-20

**Authors:** Taija Puranen, Kaija Hiltunen, Hannu Kautiainen, Merja H. Suominen, Karoliina Salminen, Päivi Mäntylä, Hanna-Maria Roitto, Kaisu H. Pitkälä, Riitta K. T. Saarela

**Affiliations:** 1 Social Services, Health Care and Rescue Services Division, Development Support, City of Helsinki, P.O. Box 6008, 00099 Helsinki, Finland; 2 Department of General Practice and Primary Health Care, University of Helsinki, Helsinki, Finland; 3 Department of Oral and Maxillofacial Diseases, Faculty of Medicine, University of Helsinki, Helsinki, Finland; 4 Primary Health Care Unit, Kuopio University Hospital, Kuopio, Finland; 5 Folkhälsan Research Center, Helsinki, Finland; 6 Institute of Dentistry, University of Eastern Finland, Kuopio, Finland; 7 Oral and Maxillofacial Diseases, Kuopio University Hospital, Kuopio, Finland; 8 Department of Medicine, University of Helsinki, Helsinki, Finland; 9 Social Services, Health Care and Rescue Services, Helsinki Hospital, Helsinki, Finland; 10 Population Health Unit, Finnish Institute for Health and Welfare, Helsinki, Finland; 11 Unit of Primary Health Care, Helsinki University Hospital, Helsinki, Finland; 12 Social Services, Health Care and Rescue Services Division, Oral Health Care, City of Helsinki, Helsinki, Finland

**Keywords:** Long-term care, Oral frailty, Health-related quality of life, Nutrient intake, Survival

## Abstract

**Purpose:**

We evaluated oral frailty (OFr) and its association with health-related quality of life (HRQoL), energy and protein intake, and survival among older long-term care residents.

**Methods:**

This cross-sectional study with a 3-year follow-up for survival assessed 349 residents in long-term care facilities (73% female, mean age 82 years). We defined OFr with six signs (dry mouth, food residue on oral surfaces, unclear speech, inability to keep mouth open or pain expression during the clinical oral examination, diet pureed/soft) and OFr severity was categorized as Group 1, (mild) = 0–1 signs, Group 2 (moderate) = 2–4 signs, and Group 3 (severe) = 5–6 signs. We measured HRQoL with 15D instrument, and energy and protein intake by a 1- to 2-day food record. Mortality was retrieved from central registers on March 2021.

**Results:**

Of the residents, 15% had 0–1, 67% 2–4 and 18% 5–6 OFr signs. HRQoL decreased linearly from Group 1 to Group 3. OFr correlated with such dimensions of HRQoL as mobility, eating, speech, excretion, usual activities, mental function, and vitality. We found no association between OFr categories and energy and protein intake. Survival decreased linearly from Group 1 to Group 3.

**Conclusions:**

OFr was common among older long-term care residents and OFr severity predicts poorer outcomes. The six oral signs denoting OFr may be used at the bedside to screen residents at risk for OFr.

## Key summary points

**Aim:**

We evaluated oral frailty (OFr) with six signs and its association with health-related quality of life (HRQoL), energy and protein intake, and survival among long-term care facilities.

**Findings:**

HRQoL decreased linearly with increasing number of OFr signs. OFr was not associated with energy and protein intake, and lower OFr category significantly predicted better survival.

**Message:**

The six signs of OFr used in this study are easy to employ in long-term care at the bedside. Oral health of older residents in long-term care facilities should be given more attention.

## Introduction

Oral health is closely linked to quality of life of older adults and oral health problems are associated with greater incidence of disability [1, 2]. There is no consensus of the definition of oral frailty (OFr), poor oral health or oral hypofunction regarding signs, symptoms, or diagnostic methods [3–6]. Tanaka et al. determined poor oral status by the number of natural teeth, chewing ability, articulatory oral motor skill, tongue pressure, subjective difficulties in eating and swallowing difficulties, and defined OFr when ≥ 3 of the six measures were present [4]. Minakuchi et al. divided oral function into four stages such as healthy state, oral frailty, oral hypofunction and oral dysfunction [6]. Minakuchi et al. defined OFr as a condition with signs or symptoms specified as decreased articulation, slight choking or spillage while eating, and an increased number of unchewable foods. Oral hypofunction was defined as the state when ≥ 3 of these diagnostic criteria are met [6]. Hiltunen et al. suggested that six oral signs (dry mouth; food residue on surface of teeth, mucosa, or dentures; unclear speech; inability to keep mouth open; oral painfulness; diet of pureed or soft food) may be used to determine OFr syndrome [7]. This study found a significant linear relationship between OFr severity and Fried’s frailty phenotype; participants with more OFr signs more often suffered from frailty, dementia, malnutrition and required help with daily activities than those with fewer signs [7].

Poor oral health status has been associated with physical frailty, sarcopenia and mortality [3, 4]. In addition, frail older adults have poorer oral function as measured by the number of teeth, occlusal force, masseter muscle thickness, and oral diadochokinesis [8]. Oral symptoms, such as sensitive teeth, toothache, bleeding gums, dry mouth, and loss of natural teeth or ill-fitting dentures are associated with poorer oral health-related quality of life (OHRQoL) [1]. Oral health problems and dysphagia may alter food choices, leading to diminished food intake, poor quality diet, and malnutrition [3, 9–12].

Previous studies have explored the association between oral health as measured by the number of teeth or occluding pairs and various oral symptoms with nutritional status, OHRQoL, sarcopenia, frailty, and mortality [1, 3, 4, 8]. However, less is known about the association between OFr and general health-related quality of life (HRQoL), energy and protein intake, and survival among long-term care residents. The aim of this study was to investigate the association between OFr defined by six signs defined by Hiltunen et al. [7] and its association with HRQoL, energy and protein intake, and survival among long-term care residents. The above-mentioned OFr assessment method is practical in the assessment of older adults with multimorbidity and cognitive disability living in long-term care. In addition, nurses can assess the residents' OFr status during daily care without special aids [7].

## Materials and methods

**Fig. 1:**
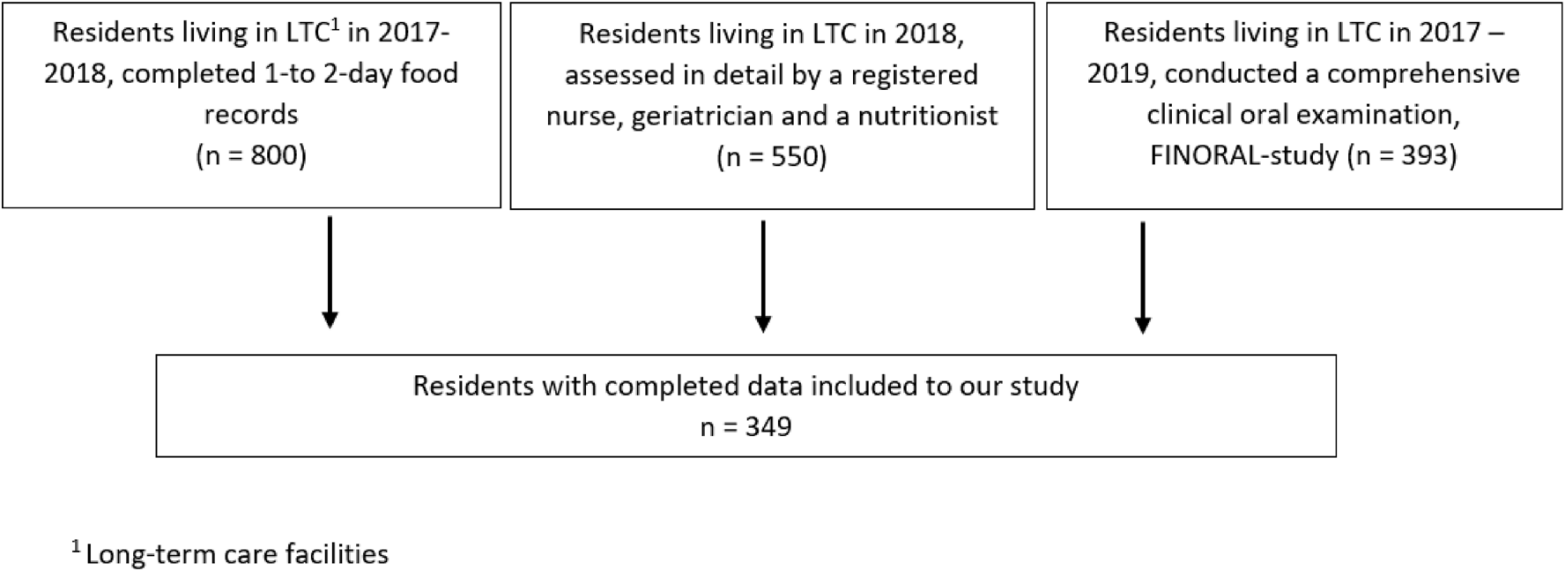
The flow chart of the study

This study is part of a larger project exploring the nutritional status and quality of nutritional care among long-term care residents in Helsinki, Finland [13]. In 2018, 550 residents were assessed in detail by a registered nurse, geriatrician, and a nutritionist. The residents’ demographic factors, nutrition, health and functional status, and HRQoL were assessed. The nutritionist organized a detailed assessment of 1- to 2-day food records for 800 residents. Furthermore, two dentists conducted a comprehensive clinical oral examination of 393 residents of the Finnish Oral Health Studies in Older Adults (FINORAL) study [7, 14]. These three databases were combined and altogether 349 participants had complete data for all the items required for this study (Fig. 1).

In 2017–2019, two qualified and calibrated dentists conducted a comprehensive clinical oral examination of 393 participants of the FINORAL study [7, 14]. They performed clinical oral examinations in long-term care settings with participants lying on their bed or sitting on a chair during the oral examination. The dentists were equipped with loupes (Merident Optergo MO Ultralight Flip-up), an attached headlamp (Merident Optergo DeLight LED), and normal sets of sterile dental instrumentation. Dentists registered the following five signs of OFr: dryness of the mouth (mirror sticks to buccal mucosa or tongue, frothy saliva, glassy appearance of oral palate lobulated/fissured tongue); presence of food residues (on surface of teeth, on surface of oral mucosa, or on surface of or under removable dentures); unclear speech; inability to keep mouth open during the examination; painfulness (expression of pain during oral examination) [7]. The use of pureed or soft food diet, which was the sixth OFr sign, was reported by nurses [7]. These six OFr signs were dichotomized as yes/no. Residents were assigned to groups based on the number of OFr signs: Group 1 (mild OFr) 0 or 1 sign, n = 52; Group 2: (moderate OFr), 2–4 signs, n = 235; Group 3 (severe OFr), 5–6 signs, n = 62.

Nurses assessed residents’ dependence in functional status by the Clinical Dementia Rating (CDR) “Personal care item” (0–0.5 = totally independent; 1 = needs prompting; 2 = requires assistance in dressing, personal hygiene, and keeping of personal belongings; 3 = being dependent in activities of daily living) [15]. Diagnoses were retrieved from medical records. Residents’ cognition was assessed by the Mini-Mental State Examination (MMSE) [16]. Comorbidity was calculated for each resident using the Charlson Comorbidity Index (CCI) [17]. Medical history and current medications were retrieved from medical records. The use of oral health care services was assessed with the question: “When was the latest oral examination performed by a dentist or a dental hygienist?” (1 = less than 1 year ago; 2 = 1–3 years ago; 3 = more than 3 years ago).

Residents’ HRQoL was measured using the 15D quality of life instrument [18]. The 15D is a generic, comprehensive, standardized measure of HRQoL that can be self-administered or proxy-rated. We used it both as a profile and as a single index score measure. The 15D consists of 15 dimensions of health (mobility, vision, hearing, breathing, sleeping, eating, speech, excretion, usual activities, mental function, discomfort and symptoms, depression, distress, vitality, and sexual activity) with five ordinal levels (best level = 1, worst level = 5). The single index score of the 15D instrument represents overall HRQoL on a scale from 0 to 1 (1 = full health, 0 = dead). A minimum clinically important change/difference in the 15D score has been estimated to be ± 0.015 [19].

Residents’ nutritional status was assessed using the Mini Nutritional Assessment (MNA) [20]. Scores of < 17, 17–23.5, and 24–30 indicate malnutrition, risk of malnutrition, and normal nutritional status, respectively. A detailed 1- to 2-day food record was completed, and energy and protein intake was determined. Nurses following the residents’ daily care were instructed to record all the foods and beverages consumed by the resident. Nurses estimated portion sizes by household measures. AivoDiet dietary software (version 2.2.0.0, Aivo Oy, Turku, Finland) was used to analyse the food records. The AivoDiet software includes the Fineli Food Composition Database Release 16 (2013). The database includes recipes for typical Finnish mixed dishes that are usually served in long-term care. For prepacked products, the exact brand and product name was given. A suitable recipe was used during data entry. The use of oral nutritional supplements (ONS) was assessed (yes/no). ONSs were included in the daily energy and protein intake. Information on 3-year mortality was retrieved from central registers in March 2021.

The local ethics committee of the Helsinki University Hospital approved the study. Informed consent was acquired from all participants or from their closest proxies in case of moderate-to-severe dementia.

### Statistical analysis

Data are expressed as mean and standard deviation (SD) or counts with percentages. The linearity across the three OFr groups was evaluated using the Cochran-Armitage test, ordered logistic regression, and analysis of variance with an appropriate contrast (orthogonal). A bootstrap-type test was used in case of violation of assumptions (e.g., non-normality). Correlation coefficients between OFr as a continuous variable and dimensions of 15D were calculated by using Spearman’s rank correlation method. Kaplan–Meier curves were used to estimate the survival in the three OFr groups; linearity was evaluated by using the log-rank test for trend. Adjusted survival curves were based on a stratified Cox model using baseline age, gender, and Charlson Comorbidity Index as covariate. The normality of variables was evaluated graphically and by using the Shapiro–Wilk *W* test. Stata 17.0 statistical package (StataCorp LP, College Station, TX, USA) was used for the analysis.

## Results

Table 1 shows participant characteristics grouped according to the OFr categories. Among long-term care residents, 15% had 0–1, 67% 2–4 and 18% 5–6 OFr signs. Mean age was 82 years, 73% were female, and 44% had a low level of education (< 8 years). The proportion of females increased linearly from Group 1 (mild OFr) to Group 3 (severe OFr) (p = 0.014). The residents in Group 3 more often exhibited dependence in activities of daily living (ADL) (p = < 0.001), had dementia diagnosis (p = 0.007), and had lower MMSE scores (p < 0.001) than residents in Group 1 or 2. Although comorbidity according to CCI did not differ between the OFr groups, the mean number of medications decreased linearly from Group 1 (10.0) to Group 3 (7.3) (p < 0.001). Of the participants, thirty-eight percent had undergone an oral examination within the past year and approximately one-fifth of the residents had their previous oral examination more than 3 years ago. There was no significant difference between the OFr in the frequency of oral examinations.

**Table 1:** Characteristics, health-related quality of life, nutrition, and survival of older residents living in long-term care facilities by Oral Frailty categories

	Group 1 0–1 sign^a^ N = 52	Group 2 2–4 signs N = 235	Group 3 5–6 signs N = 62	P for linearity
Age, years, mean (SD)^a^	82 (9)	82 (8)	82 (9)	0.92
Females, n (%)	32 (62)	171 (73)	51 (82)	0.014
Education < 8 years, n (%)	27 (57)	99 (47)	27 (47)	0.34
Functioning and health				
Dependence in ADLs^a^, n (%)	38 (73)	205 (91)	61 (98)	< 0.001
Dementia, n (%)	35 (67)	171 (73)	55 (89)	0.007
MMSE^a^, mean (SD)	16.5 (6.5)	13.7 (7.2)	8.7 (6.7)	< 0.001
Charlson Comorbidity Index, n (%)	2.0 (1.3)	1.9 (1.2)	1.7 (1.1)	0.11
Number of medications, mean (SD)	10.0 (3.5)	9.1 (3.6)	7.3 (3.6)	< 0.001
Previous oral examination performed by a dentist/dental hygienist, n (%)				0.80
< 1 year ago	21 (44)	88 (42)	24 (41)	
1–3 years ago	11 (23)	78 (37)	21 (36)	
≥ 3 years ago	16 (33)	43 (21)	14 (24)	
	0.701 (0.114)	0.624 (0.126)	0.538 (0.083)	< 0.001
Health-related quality of life				
15D score, mean (SD)	0.701 (0.114)	0.624 (0.126)	0.538 (0.083)	< 0.001
Nutrition				
MNA^a^, n (%)				< 0.001
24–30 (Normal nutritional status)	19 (39)	44 (21)	2 (4)	
17–23.5 (At risk of malnutrition)	29 (59)	138 (66)	39 (68)	
< 17 (Malnutrition)	1 (2)	26 (13)	16 (28)	
Energy intake, kcal (SD), mean total	1726 (384)	1660 (378)	1617 (375)	
Energy intake, kcal (SD), female	1671 (383)	1617 (368)	1562 (354)	
Energy intake, kcal (SD), male	1824 (376)	1779 (383)	1868 (377)	0.88
Protein intake, g, (SD) mean, total	0.86 (0.28)	0.88 (0.27)	0.94 (0.35)	
Protein intake, g/bodyweight (kg), mean (SD), female	0.90 (0.32)	0.89 (0.29)	0.94 (0.37)	
Protein intake, g/bodyweight (kg), mean (SD), male	0.78 (0.19)	0.84 (0.23)	0.93 (0.27)	0.10
Use of oral nutritional supplements, n (%)	5 (10)	34 (15)	21 (34)	< 0.001
Survival				
Survival at end of follow up, % (95% CI)	44 (30–58)	30 (23–37)	19 (9–31)	< 0.001

*SD* standard deviation, *ADL* activities of daily living measured by Clinical Dementia Rating (CDR) scale “personal care” score ≥ 3, *MMSE* Mini Mental State Examination, *MNA* Mini Nutritional Assessment

^a^Dry mouth, diet of pureed or soft food, food residue on oral surfaces, unclear speech, inability to keep mouth open, painfulness

**Fig. 2:**
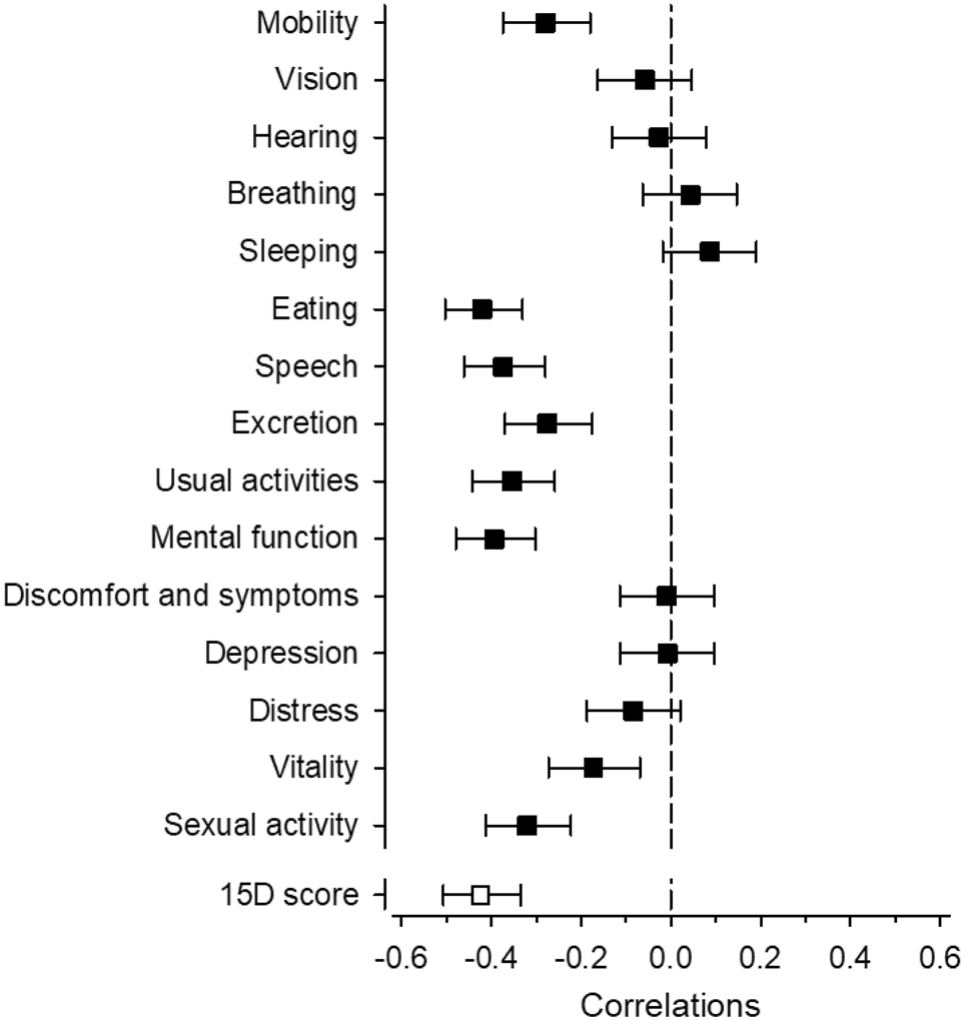
Spearman’s correlations with 95% confidence intervals between number of oral frailty signs and dimensions of health-related quality of life (HRQoL) measured by 15D

HRQoL according to 15D decreased linearly as the severity of OFr increased (p < 0.001). The participants’ crude mean 15D scores were 0.70 (Group 1), 0.62 (Group 2), and 0.54 (Group 3) (Table 1). Eight dimensions of 15D (mobility, eating, speech, excretion, usual activities, mental function, vitality, and sexual activity) and total 15D score correlated negatively with the number of OFr signs (Fig. 2).

The proportion of residents who were malnourished or at risk for malnutrition increased from Group 1 to Group 3 as measured with the MNA. There were no significant differences in energy intake or protein intake (g/bodyweight in kg) between the OFr groups. The proportion of residents who received ONSs increased linearly from Group 1 to Group 3 (p < 0.001).

**Fig. 3:**
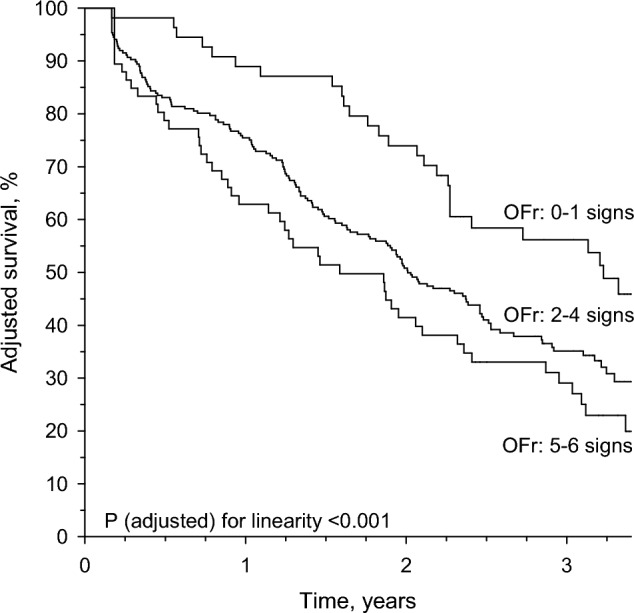
Kaplan–Meier survival curves of residents in long-term care facilities according to OFr categories (Group 1 = 0–1 signs, Group 2 = 2–4 signs, and Group 3 = 5–6 signs) adjusted for sex, age, and Charlson comorbidity index. Adjusted survival (%) decreased when the number of OFr signs increased

Sixty-four percent (n = 224) died during the 3-year follow-up period. Kaplan–Meier curves decrease linearly from Group 1 to Group 3 during three-year follow-up adjusted for sex, age, and CCI (Fig. 3).

## Discussion

Among long-term care residents, 15% had mild (0–1 signs), 67% moderate (2–4 signs) and 18% severe (5–6 signs) OFr. The proportion of residents who needed help with daily activities, had dementia diagnosis and lower MMSE increased linearly from Group 1 to Group 3. HRQoL decreased linearly with increasing number of OFr signs. OFr correlated with such dimensions of 15D as mobility, eating, speech, excretion, usual activities, mental function, and vitality. The proportion of residents who were malnourished or those at risk for malnutrition increased from Group 1 to Group 3. We found no association between OFr groups and energy and protein intake, but the proportion of residents who received ONSs increased from Group 1 to Group 3. Survival decreased linearly from Group 1 to Group 3.

The strengths of this study are the well-validated and widely used assessment tools and a structured questionnaire validated in previous nutritional studies in Helsinki in 2003–2011 [21]. Two qualified and calibrated dentists performed thorough clinical oral examinations and food records were collected by nurses who were trained for this procedure by a nutritionist. In institutional settings, food records with direct observation have been considered more appropriate than food frequency questionnaires [22]. HRQoL assessed with the 15D instrument can been completed by proxy and may thus also be used for long-term care residents with cognitive decline. However, proxies may overestimate the impact of cognitive decline on HRQoL compared to participants [23], which may be a limitation of our study. Another limitation is the cross-sectional nature, which makes it impossible to draw causal relationships between OFr, nutritional status and HRQoL, for example. However, our study had a longitudinal design concerning survival. In oral examination, as the participants were lying on their bed or sat on a chair, the accuracy of the examination does not correspond to that of any examination performed in a dentist’s office with better visibility. The OFr definition has only been used in one study before, which may be considered as a limitation [7]. Our definition differs e.g. from Tanaka’s, which includes signs such as tongue pressure, chewing ability and number of teeth [4] and Morley’s suggestion to screen OFr with the D-E-N-T-A-L or EAT-10 questionnaires [24].

Our study showed that HRQoL decreased linearly as the number of OFr signs increased. We used a generic 15D instrument to measure HRQoL [18]. To the best of our knowledge, OFr and its association with general HRQoL has not been investigated. Previous studies have investigated OHRQoL; Porter et al. used the Oral Impacts on Daily Performances tool and found that self-reported oral problems were associated with poorer OHRQoL [1]. Klotz et al. measured OHRQoL with the Geriatric Oral Health Assessment Index and revealed that factors associated with OHRQoL included number of teeth, prosthetic status, and denture-related treatment needs [25]. de Oliviera et al. measured HRQoL with SF-12 and did not find a significant association between HRQoL and self-perceived oral health among institutionalized older adults [26]. However, poor oral hygiene and burden of oral symptoms are associated with poorer HRQoL measured with 15D instrument [13, 27]. The 15D dimensions of mobility, eating, speech, excretion, usual activities, mental function, vitality, and sexual activity correlated with the number of OFr signs. Difficulties in mobility and in usual activities are linked to general frailty. Frailty is associated with an increased risk for disability, falls, hospitalisation and death, impaired HRQoL and malnutrition among older adults [28–31]. OFr signs such as dry mouth and painfulness, may complicate eating and lead to the need for soft and pureed food, which may increase the risk for malnutrition [32].

We found no significant difference in energy or protein intake between the groups, although the proportion of malnourished increased from Group 1 to Group 3. This may be explained by the use of ONSs; residents with severe OFr more often received ONSs than those with mild OFr. Thus, nurses recognize malnutrition and accordingly administer ONSs to those residents, which may dilute our results. ONSs are recommended to enhance the nutrition (e.g., energy and protein intake) of older adults who are malnourished or at risk for malnutrition and for those with signs of dysphagia, chewing problems, or both [33]. The mean protein intake of our participants was low compared to the recommendations of at least 1 g/kg body weight daily [33], which is in line with the previous study [34]. Even greater amounts of protein are recommended (1.2–1.5 g/kg body weight/day) for older people with acute or chronic diseases [35].

Our study suggests OFr severity predicts diminished survival. This is in line with the studies that have reported an accumulation of oral problems increases the risk for mortality [4, 36, 37]. Hiltunen et al. found a strong association between OFr severity and Fried’ s frailty phenotype in the same study population as ours [7], and furthermore frail older adults have the highest risk for mortality [38]. Some studies have reported the association between various oral problems and mortality among institutionalized [39], hospitalized [40], and community-dwelling older adults [4, 37]. However, these studies used different instruments to measure oral health, such as Revised Oral Assessment Guide (categories of voice, lips, mucous membranes, tongue, gums, teeth/dentures, saliva, and swallowing sensation) [39], Oral Health Assessment Tool (categories of lips, tongue, gums and tissues, saliva, natural teeth, dentures, oral cleanliness, dental pain) [40], and number of teeth, swallowing disability, oral dryness, and poor oral hygiene [37].

The purpose of this study was to test previously proposed OFr signs [7] and their association with HRQoL, nutrient intake, and survival. The signs were selected according to previous studies [3, 4, 41–45]. The definition of oral frailty (OFr) is mainly based on weakening of the oral muscles and on difficulties in chewing, eating, speaking and swallowing [4]. The problem goes both ways, as swallowing difficulties are associated with chewing difficulties due to reduced mastication and often co-occur in institutionalized residents. Hiltunen et al. and our study used the following: dry mouth, food residue on oral surfaces, unclear speech, inability to keep mouth open, pain expression, diet pureed/soft) [7]. Our participants were residents in long-term care facilities, in need of constant care and majority had dementia. Questionnaires or complicated assessments are not possible among this group, and therefore we aimed to test OFr signs easily evaluated in clinical setting, even by nurses. We used dry mouth as one of the signs. Dry mouth is considered a sign of general fatigue, and it is associated with chewing and swallowing difficulties, dehydration, dementia, and unclear speech [46], and previously considered as a sign of OFr [24]. Furthermore, we used the presence of food residues, unclear speech, inability to keep mouth open, and food softness as a surrogate signs for muscle weakness [7]. Masticatory muscle weakness is related to muscle size, function and ability to maintain muscle strength [8], but measurements for occlusal force, muscle thickness or kinetics, however, were not possible with our study participants. In addition, we evaluated pain with the pain expressions of sounds, turning the head and partial or total refusal of further examination that the ‘Orofacial Pain Scale for Non-Verbal Individuals’ has recommended [47, 48].

Our study population was prone to oral and general health problems; majority had more than two OFr signs, most residents needed help in daily activities, suffered from dementia, had multiple comorbidities and medications, and only 38% of participants had undergone an oral examination within the past year. Therefore, oral health should be given more attention. Some studies indicate that oral treatment according to individual needs may improve quality of life and oral intervention programs may reduce the risk of mortality among long-term care residents [49, 50]. Our results should be confirmed in the larger populations, and we need intervention studies to investigate whether oral health interventions at the earlier state of life have effects on OFr, quality of life and survival in old age. In addition, future studies should investigate the predictive value of OFr for quality of life, general well-being and survival.

## Conclusions

OFr measured with six signs (dry mouth, food residue on oral surfaces, unclear speech, inability to keep mouth open, painfulness, diet of pureed or soft food) may be used to evaluate OFr syndrome and its severity. OFr severity was linearly associated with diminished HRQoL and survival. The six signs used in this study are easy to employ in long-term care at the bedside.
