# Correction: Relationship between oral frailty, health-related quality of life, and survival among long-term care residents

**DOI:** 10.1007/s41999-023-00886-8

**Published:** 2023-10-24

**Authors:** Taija Puranen, Kaija Hiltunen, Hannu Kautiainen, Merja H. Suominen, Karoliina Salminen, Päivi Mäntylä, Hanna Maria Roitto, Kaisu H. Pitkälä, Riitta K. T. Saarela

**Affiliations:** 1 Social Services, Health Care and Rescue Services Division, Development Support, City of Helsinki, P.O. Box 6008, 00099 Helsinki, Finland; 2 Department of General Practice and Primary Health Care, University of Helsinki, Helsinki, Finland; 3 Department of Oral and Maxillofacial Diseases, Faculty of Medicine, University of Helsinki, Helsinki, Finland; 4 Primary Health Care Unit, Kuopio University Hospital, Kuopio, Finland; 5 Folkhälsan Research Center, Helsinki, Finland; 6 Institute of Dentistry, University of Eastern Finland, Kuopio, Finland; 7 Oral and Maxillofacial Diseases, Kuopio University Hospital, Kuopio, Finland; 8 Department of Medicine, University of Helsinki, Helsinki, Finland; 9 Social Services, Health Care and Rescue Services, Helsinki Hospital, Helsinki, Finland; 10 Population Health Unit, Finnish Institute for Health and Welfare, Helsinki, Finland; 11 Unit of Primary Health Care, Helsinki University Hospital, Helsinki, Finland; 12 Social Services, Health Care and Rescue Services Division, Oral Health Care, City of Helsinki, Helsinki, Finland

**Correction: European Geriatric Medicine** 10.1007/s41999-023-00859-x

In the original version of this article, the given and family names of [AUTHOR NAME] were incorrectly structured.

Correct is: Hanna Maria (first name) Roitto (last name).

The original article has been updated.

